# Neuropsychiatric Symptoms of Alzheimer's Disease and Caregiver Burden

**DOI:** 10.3389/fneur.2022.877143

**Published:** 2022-07-29

**Authors:** Behnam Iravani, Elaheh Abdollahi, Fatemeh Eslamdoust-Siahestalkhi, Robabeh Soleimani

**Affiliations:** Kavosh Cognitive Behavior Sciences and Addiction Research Center, Department of Psychiatry, School of Medicine, Guilan University of Medical Sciences, Rasht, Iran

**Keywords:** neuropsychiatric symptoms, Alzheimer's disease, caregiver burden, Alzheimer's, caregiver

## Abstract

**Introduction/Objectives:**

In addition to cognitive decline, one of the most important problems for caregivers of patients with Alzheimer's is neuropsychiatric symptoms (NPS). This study aimed to evaluate the NPS in patients with Alzheimer's disease (AD) and investigate its relationship with caregiver burden (CB).

**Methods:**

In a cross-sectional study of 85 patients with AD referred to Shafa Hospital in Rasht and their caregivers in 2020, information was collected using a demographic questionnaire, Neuropsychiatric Inventory Questionnaire (NPI-Q), and the Caregiver Burden Inventory (CBI). Data were analyzed by Spearman correlation, *t*-test, and linear regression, with SPSS version 22.

**Results:**

The mean age of the patients and their caregivers were 74.95 ± 8.87 years and 43.98 ± 11.38 years, respectively. The mean total score of NPS in patients with AD was 44.25 (0–144) and the mean CB score was 36.27 (0–96), which was a moderate level. According to the results, 91% of patients had apathy, while happiness/euphoria was reported as the most uncommon symptom. In addition, there was a significant relationship between the score of NPS and CB (*r* = 0.542, *P* < 0.0001), as well as all its sub-components, time-dependence burden with more correlation (*r* = 0.509, *P* < 0.0001), and social burden with less correlation (*r* = 0.352, *P* < 0.001). NPS, hallucination, aberrant motor behavior (AMB), delusion, and depression were most correlated with CB. Also, the mean score of CB was significantly higher in women than in men (*P* = 0.045). Living in a rural area had a significant relationship with NPS score (*P* = 0.026). Also, linear regression showed that with increasing 1 year of patients' age, the mean score of patient's NPS decreased by 0.374 (*P* = 0.048).

**Conclusion:**

Neuropsychiatric symptoms, especially hallucination, aberrant motor behavior (AMB), delusion, and depression were associated with caregiver burden. Apathy was the most common symptom in patients with AD.

## Introduction

Increasing the number of older people is one of the important issues in the twenty-first century (1). An increase in the rate of aging is inevitable, and of course, Alzheimer's disease (AD) is the most common cause of dementia in older adults (2). Around the world, over 50 million people are currently living with AD-related dementia, and this number could also rise to 152 million by 2050 (World Alzheimer's Report 2019) (3). AD is a progressive and irreversible brain disorder (4) that affects ~300–450 thousand Iranians (5). In a recent study in 2019, the prevalence of AD in Iran was estimated at 2.3% (6). Iran is one of the leading and increasing aging populations in the world. As a global health problem, concerns raises about the pace of future economic growth, performance and financial integrity of health care systems, and pensions and the health and wellbeing of the elderly (7).

In the vast majority (>80%) of the patient with AD, in addition to cognitive decline, the onset of functional and behavioral deficits, which are known as neuropsychiatric symptoms (NPS) (8), can increase the caregiver burden (CB) (9). The coincidence of NPS and mild cognitive impairment (MCI) are associated with an increased risk of dementia (10). As a result, subtle NPS is one of the first preclinical signs of AD (8, 11, 12). So, detection and treatment of NPS, especially apathy, in early dementia can delay the progression of the disease (13).

NPS is often overlooked, especially when the patient has a cognitive impairment in dementia (14, 15), and is the major contributor to CB (16). They exacerbate functional deficits and disabilities in social skills, accelerate cognitive decline in patients (12), increase CB, distress, and adverse outcomes, such as depression and reduced quality of life. Also, they can cause further neuropathological symptoms (17, 18).

The study by Mavounza et al. (19) found that the frequency/severity of NPS in patients and the emotional distress of caregivers had a positive association. In a review study about CB and NPS presence in patients, results showed that caregivers of patients with AD experience more stress than caregivers of older adults in lack NPS. Moreover, the presence of NPS in patients was related to more CB (16). Yu et al. (20) found that more severe AD and more caregiving time were associated with more CB.

In a study in Iran, the level of CB in most caregivers was moderate to severe. The high level of CB was significantly associated with gender, patient education, and patient's level of dependence on daily activities (21). In another study in 2010, the level of general mental health problems was moderate to severe in about 75% of female caregivers of patients with AD (22).

Inadequate knowledge about Alzheimer's disease and poor premorbid relationships with patients can predict CB (16). These characteristics are the only face of the Rubik's Cube. Many others that can be predictors or protective factors of this burden should be evaluated to solve this puzzle.

Patients' characteristics, such as AD's severity, MCI, the decline in or loss of functional activities of daily living, and demographic features (23, 24) and caregivers' characteristics (25) have been investigated as a predictor of NPS in many studies.

Caring for a person with Alzheimer's is more stressful than other chronic disorders (26). Sleep in Alzheimer's caregivers has always been of low quality and quantity. Caregivers are often woken up overnight by their patients, and as a result, they find that they will have a better sleep when they rest from caring for a person with Alzheimer's (27). Most caregivers are family, friends, or neighbors. This means that caregivers may be older than patients and not physically and mentally healthy. Although young caregivers especially children of patients have many responsibilities in middle age and in addition to their relationship with the patients, they lose their free time and have problems in their jobs, and experience anxiety and depression. Therefore, dealing with AD, in general, can harm the physical, psychological, emotional health, and social and economic life of caregivers (28), young and old alike.

Due to the high prevalence of Alzheimer's disease as the most common cause of dementia in the world and the large number of people involved in the care of these people and limited studies in this field in our country, this study aimed to evaluate neuropsychiatric symptoms in patients with AD and its relationship with CB.

## Methods

### Study Design

In this cross-sectional study, patients with AD who had been referred to Shafa Hospital of Rasht in 2020 and their caregivers were included in the study after obtaining informed consent and ensuring that the inclusion criteria were met.

Inclusion criteria for patients were confirming the AD diagnosis by one of the hospital psychiatrists. Caregivers should not have a history of cognitive impairment, traumatic brain injury, or neurological disorders and take care of patients for at least 1 night a week for 1 month.

To reduce the coronavirus disease 2019 (COVID-19) spread, the sample information was collected through their files in the hospital. Most of the questionnaires were filled out *via* telephone or email. Some of them were done in a private area at the center with full compliance with the health protocols. The questionnaires were given to 89 caregivers (58 people virtually and 21 physically). Four out of all participants were excluded from the study due to incomplete questionnaires, so 85 individuals were included.

### Assessment

Demographic questionnaire, Neuropsychiatric Inventory (NPI) (29), and Caregiver Burden Inventory (CBI) (30) were used for the measure of the study.

#### Neuropsychiatric Inventory Questionnaire

This scale is a caregiver-based questionnaire, in which the carer indicates the presence or absence of neuropsychiatric symptoms in the patient during the last few weeks, including 12 neuropsychiatric symptoms: delusions, hallucinations, agitation/aggression, dysphoria/depression, anxiety, euphoria/elation, apathy/indifference, disinhibition, irritability/lability, aberrant motor behaviors (AMB), night-time behavioral disturbances, and appetite/eating disturbances. After answering the screening question by the caregivers, they answer the sub-questions related to each behavior, and then, indicate the frequency (1 rarely, less than once per week to 4 very often, once, or more per day) and severity (1 mild to 3 severe) of it. To assess the caregiver's distress score, after determining the severity and frequency of the symptom, we ask the caregiver: “How much do these symptoms cause you discomfort?” Caregiver stress is scored between 0 (no distress) and 5 (extreme distress). For each symptom, the total score is calculated by the equation frequency × severity. The total NPI scores are within 0–144. The original instrument had good validity and reliability (29). The internal consistency by Cronbach's α was 0.9 and concurrent validity was obtained between 0.3 and 0.9 (*P* < 0.05) in the Persian version of this scale (31).

#### The Caregiver Burden Inventory

This scale consists of 24 open-ended and fixed choice questions. The instrument assesses the CB (4 nearly always to 0 never), with each of the following 5 dimensions of CB: time-dependence, developmental, physical, social, and emotional dimensions. Total scores can range from 0 to 96, and higher scores indicate greater CB. The total CBI score between 0–15 is considered to be mild, 16–47 moderate, and 48–96 severe. Cronbach's alpha for the scale was 0.9, and the test and retest scores were 0.8 (32).

Interviewees also answered a series of demographic questions. The interview took approximately 1 h.

### Human and Animal Rights

No animals were used in this study. This study was conducted based on the ethical principles for medical research involving human subjects by the World Medical Association Declaration of Helsinki. It was approved by the Ethical Committee of Guilan University of Medical Sciences (IR.GUMS.REC.1399.177).

### Statistical Analysis

The demographic feature, prevalence, and incidence of NPI and CBI scores were assessed in patients and caregivers using *t*-test, analysis of variance (ANOVA) test, and correlation test (Bivariate analysis with SPSS version 22). Multiple regression analysis and simple correlation analyses were used to demonstrate the factors affecting the CB, according to the presumptions of parametric tests in the present study. To investigate the relationship between two continuous variables, the score of NPS and CB, due to the lack their normality, they were measured by Shapiro-Wilkes's test (*P* < 0.0001). The Spearman correlation coefficient has been used to determine the relationship between the score of NPS and CB.

## Results

A total of 85 patients with AD and their caregivers participated in the study. The mean age of patients and caregivers was 74.95 ± 8.87 and 43.98 ± 11.38 years, respectively, and the mean time of AD in patients participating in this study was 5.09 ± 3.26 years. The median number of caregivers was 3 (2–4), and the mean duration of caring by caregivers was 40.26 ± 34.22 months. Among the drugs used by the patients, most of them were acetylcholinesterase inhibitors (AchEs) (82.4%), Benzodiazepines (72.9%), and selective serotonin reuptake inhibitors (SSRIs) (49.4%) respectively. Demographic and clinical characteristics of patients and caregivers are displayed in Table 1.

**Table 1:** Demographic and clinical characteristics of participants.

**Characteristics**	**Patients**		**Caregivers**	
	***N***	**%**	***N***	**%**
**Gender**				
Female	59	69.4	49	57.6
Male	26	30.6	36	42.4
**Place of residence**				
Urban	62	72.9	76	89.4
Rural	23	27.1	9	10.6
**Marital status**				
Single	25	30.1	18	21.2
Married	58	59.9	67	78.8
**Employment status**				
Unemployed/Homemaker	44	51.8	34	40.0
Self-employed	23	27.1	26	23.5
Employee/worker	18	21.1	25	36.5
**Educational level**				
Illiterate	28	34.6	0	0.0
< Diploma	28	34.7	9	10.7
Diploma	20	24.7	25	29.4
Bachelor's degree or above	5	6.2	51	60.1
**Economic status**				
Low	28	33.3	13	15.3
Middle	42	50.0	53	62.4
High	14	16.7	19	22.4
**Relationship with the patient**				
Spouse	–	–	4	4.8
Daughter	–	–	32	38.1
Son	–	–	33	39.3
Family	–	–	8	9.5
Etc	–	–	7	8.3
Medical disease	58	68.2	21	24.7
Psychological disease	–	–	8	9.4
Substance abuse	9	11.1	11	12.9

The mean total score of NPS in patients with AD participating in this study was 44.25 based on 12 symptoms.

Table 2 shows the prevalence, frequency, and severity of NPS in the total sample. Almost all patients in this study had apathy. Symptoms of depression, anxiety, change in appetite, and irritation/aggression have been observed to be high. The sign of happiness/euphoria has been observed with the lowest frequency.

**Table 2:** Frequency and severity of neuropsychiatric symptoms.

	***N* (%)**	**Frequency** **Mean ±SD**	**Severity** **Mean ±SD**	**Total** **Mean ±SD**
Apathy/indifference	78 (91)	2.93 ± 1.16	2.00 ± 0.94	6.58 ± 3.71
Depression/dysphoria	67 (78.6)	1.96 ± 1.30	1.70 ± 1.06	4.50 ± 3.68
Appetite and eating change	63 (73.8)	2.25 ± 1.50	1.50 ± 1.09	4.74 ± 4.03
Anxiety	62 (72.6)	1.74 ± 1.28	1.45 ± 1.01	3.54 ± 2.98
Agitation/aggression	62 (72.6)	2.08 ± 1.47	1.58 ± 1.17	4.71 ± 4.04
Sleep disturbance	57 (66.7)	1.86 ± 1.45	1.35 ± 1.06	3.85 ± 3.76
Delusions	55 (64.3)	1.85 ± 1.55	1.36 ± 1.17	4.07 ± 4.01
Disinhibition	54 (63.1)	1.63 ± 1.44	1.14 ± 1.00	2.93 ± 2.98
Irritability/lability	52 (60.7)	1.67 ± 1.43	1.39 ± 1.42	3.61 ± 3.51
Aberrant motor behavior (AMB)	46 (53.6)	1.79 ± 4.04	1.00 ± 1.04	2.71 ± 3.55
Hallucinations	36 (42.9)	1.10 ± 1.39	0.86 ± 1.08	2.26 ± 3.21
Elation/Euphoria	15 (16.7)	0.37 ± 0.91	0.29 ± 0.70	0.70 ± 1.96

*SD, standard deviation*.

According to the results, there was a statistically significant relationship between the score of NPS and CB as well as all its sub-components, by the Spearman correlation coefficient (Table 3).

**Table 3:** The relationship between the mean score of neuropsychoatric symptoms (NPS), total caregiver burden (CB) score, and sub-components of CB.

**Components scores**	**Total CB score**	**Time-dependence burden**	**Developmental burden**	**Physical burden**	**Emotional burden**	**Social burden**
Correlation coefficient	<0.542	<0.509	<0.498	<0.374	<0.408	<0.352
Significance level	<0.0001	<0.0001	<0.0001	<0.0001	<0.0001	<0.001

The relationships between each NPS with CB are presented in Table 4. According to the findings, hallucinations, AMB, delusion, and depression had the most correlation with CB, respectively. The least correlation belonged to apathy.

**Table 4:** The relationships between the mean score of CB and each NPS.

	**Mean (SD)**	***T***	**df**	***P*-value**
Hallucination	43.20 ± 08.15	2.643	82	0.010
Aberrant motor behavior (AMB)	41.22 ± 33.21	2.616	82	0.011
Delusion	41.20 ± 28.19	3.047	82	0.003
Depression	40.21 ± 11.24	3.368	82	0.001
Appetite and eating	39.22 ± 68.06	2.554	82	0.013
Irritability/lability	39.19 ± 08.72	1.560	82	0.123
Agitation/aggression	38.20 ± 77.36	1.783	82	0.078
Elation/Euphoria	38.18 ± 64.18	0.650	82	0.518
Sleep disturbance	37.22 ± 75.37	0.978	82	0.331
Disinhibition	37.19 ± 02.67	0.430	82	0.668
Anxiety	36.20 ± 80.97	1.079	82	0.284
Apathy/indifference	35.21 ± 68.67	−0.858	82	0.394

The mean CB score in caregivers was 36.27 ± 18.21, which was the moderate level. Of the five dimensions of CB, only the time-dependence burden with a mean of 11.93 ± 5.78 was higher than the mean score (scoring between 0 and 20).

In addition, among the demographic variables, the place of residence had a statistically significant relationship with the score of NPS (*P* = 0.026). The score of NPS in patients living in rural areas with a mean ± standard deviation (SD) of 42.48 ± 18.36 was significantly higher than the score of these symptoms in urban residents with an average of 33.03 ± 16.51.

Table 5 shows that apart from the caregiver age, other demographic variables of caregivers have a significant effect on CB.

**Table 5:** Descriptive and inferential results of the relationship between demographic variables and CB of caregivers of patients with Alzheimer's disease (AD).

	**Patient**			**Caregiver**		
	**Mean ±SD**	**F/t**	***P*-value**	**Mean ±SD**	**F/t**	***P*-value**
**Gender**			
Male	41.25 ± 52.13	−1.489	0.140	39.23 ± 88.27	1.973	0.045
Female	34.19 ± 05.06			31.16 ± 23.90		
**Place of residence**			
Rural	68.21 ± 48.20	−2.232	0.028	35.21 ± 19.09	−1.365	0.176
Burden	57.20 ± 18.49			45.20 ± 33.83		
**Marital status**
Married	37.21 ± 53.91	−0.635	0.527	38.21 ± 48.31	−1.959	0.047
Single	34.19 ± 28.83			28.19 ± 17.07		
**Employment status**		
Unemployed/Homemaker	35.19 ± 70.10	0.110	0.896	45.22 ± 50.47	6.125	0.003
Self-employed	38.23 ± 04.29			30.17 ± 95.41		
Employee/Retired	35.24 ± 35.37			29.18 ± 42.45		
**Economic status**
Low	47.22 ± 81.48	6.881	0.002	55.25 ± 83.25	7.718	0.001
Middle	29.18 ± 86.59			31.17 ± 28.98		
High	33.18 ± 14.49			37.20 ± 84.32		
**Medical disease**			
Yes	60.23 ± 34.05	0.046	0.964	62.22 ± 29.88	0.414	0.618
No	60.16 ± 12.65	–	–	59.20 ± 60.73	–	–
**Psychiatric disease**		
Yes	–	–	–	87.22 ± 25.83	0.432	<0.0001
No	–	–	–	57.19 ± 43.02		
**AD's treatment**			
SSRIs	64.21 ± 07.46	1.661	0.101	–	–	–
Mood stabilizers	68.24 ± 07.45	1.587	0.116	–	–	–
AChE	57.20 ± 78.07	−2.376	0.020	–	–	–
Benzodiazepine	62.22 ± 52.22	1.601	0.113			
Etc	67.20 ± 50.94	1.891	0.042	–	–	–

*Etc, other psychiatric drugs, medicinal herbs, medicinal supplements, and traditional treatments*.

**Figure 1:**
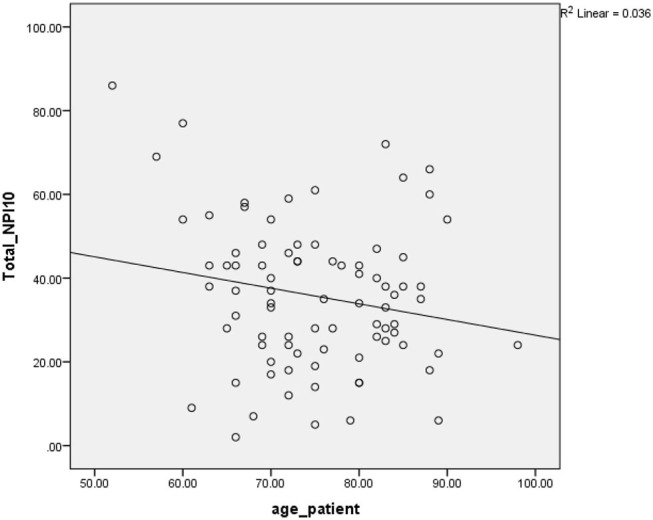
The relationship between age and NPS.

A linear regression model was used to investigate the relationship between the continuous variables of age, education, and duration of disease with the score of neuropsychiatric symptoms. A significance level of age variable with the value of 0.048 showed that with increasing 1 year of patients' age, the average score of the patient's neuropsychiatric symptoms has decreased by 0.374 units. A significance level of the fitted regression model for the education variable (*P* = 0.639) and duration of illness (*P* = 0.380) indicated that there is no significant relationship between these two factors and the score of neuropsychiatric symptoms. The distribution diagram and the fitted regression line are shown in Figure 1.

## Discussion

This study aimed to measure the NPS, as a core feature of AD and assess the CB in the caregivers. This study showed that apathy, depression, aggression, and anxiety are the most common NPSs in patients with AD. According to a systematic review and meta-analysis of the 48 original types of research in America, Europe, and Asia, the most frequent NPS was apathy and similar to our study the least common was euphoria (33). Also, in the study by Lanctôt et al. (34), Delfino et al. (35), and Chen et al. (36), apathy was increasingly recognized as an important NPS in AD.

We found that NPS was associated with CB and there was a statistically significant relationship between all sub-components of CB and NPS. The results of this study are in line with previous findings showing that the presence of NPS in people with AD is associated with both increased burden and lower quality of life in caregivers (37). Also, in the study by Chen et al., the results showed that NPI score was independently associated with mean NPI-burden score (38). Pinyopornpanish et al. (39) study in 2021 also showed that the higher the NPS score, the higher the care pressure. In addition, among neuropsychiatric symptoms, hallucination (*P* = 0.010), AMD (*P* = 0.011), delusion (*P* = 0.003), depression (*P* = 0.001), and changes in appetite/eating (*P* = 0.013) had significant association with CB. Previous studies showed different findings. In a recent study on female patients with AD, specific NPS including crying spells, anger, and aggression were associated with higher level of CB (40). In another study by Chen et al. among NPS, apathy, irritability, sleep disturbance, anxiety, and delusions were related with caregiver burden (36). According to a systematic review in 2012, it was reported that CB was negatively associated with depression, aggression, and sleep disturbances (41). In another systematic review study in 2017, irritability, agitation, sleep disturbances, anxiety, apathy, and delusion had the most relationship with CB (42). In our study apathy had the least correlation with CB. It seems that apathy with lack of motivation, emotion and poor physical activity is less annoying for caregivers than other symptoms.

Our study showed that with increasing one year of patients' age, the mean score of the NPS decreased by 0.374 score, but in the study by Okura et al. (43), the older the patient, who had more severe cognitive impairment, the greater the NPS.

We found that patients living in rural areas had higher NPS. It seems that low level of education and awareness in rural patients and caregivers might affect dealing with the disease. On the other hand, treatment may be delayed or interrupted due to difficult access to the therapist or financial problems. So, it might lead to higher NPS and CB.

The results of our study showed no significant correlation between caregiver age and CB. Some studies reported that younger caregivers had greater burden (44, 45). On the contrary, Tsai et al. (46) reported that older caregivers have much more CB; while in Pattanayak study, older caregivers perceived more spouse related burden (47). Given these contradictory results, it is not possible to make a definitive statement about the relationship between the age of the caregiver and the CB, and it seems that several other factors also play a role in this issue.

In our study, it was demonstrated that CB in females is significantly higher, especially in housewife ones. This result was in agreement with the findings of many other studies (9, 48–50). The majority of the caregivers in our sample were the children of the patients and most of them were housewives. Females have multiple roles and responsibilities in a traditionally oriented household. In addition to playing the role of mother to their children, they must take care of their older generation and live with their spouse. This condition requires a flexible personality and is normally stressful and may lead to anxiety and depression. So, female caregivers face the risk to perceive an especially higher burden. Contrary to our results, the Pinyopornpanish et al. (39) study showed that no sociodemographic variables were significantly related to CB and NPI.

The results of this study, like the study by García-Alberca (9), have shown that the duration of caregiving has a positive association with level of CB. Also, we found that by increasing caregiver education, the level of CB decreased. This result is in line with more previous studies (9, 38, 51). It seems that people with higher education have more knowledge and awareness, which can help them adapt to difficult situations.

About 9.4% of caregivers had psychiatric illness and as expected, CB was significantly higher in them. In the study by Hosseiny et al. (52), caregivers believed that caring for the elderly reduced their health and led to mental fatigue, burden of care, mental disorders, and so on. They considered caring for the elderly as a stressful experience and often found themselves unsuccessful in dealing with stress. In contrast, in a study in Spain, the majority of caregivers, who lived with the patient, had excellent mental and physical health (9). In many developed countries, there is a wide range of social support for patients with AD and their caregivers, and support groups provide a wide range of services to these individuals, thus, reducing their caregiver burden.

## Limitations

Our sampling was done from a hospital treatment center. Therefore, it is likely that our patients have more severe symptoms and more cognitive decline than patients in the community.

## Conclusion

Neuropsychiatric symptoms were associated with caregiver burden. Apathy was the most and happiness/euphoria was the least symptoms in patients with AD. The symptoms of hallucination, aberrant motor behavior (AMB), delusion, and depression had the most correlation with CB, respectively. People in rural areas had more NPS and NPS decreased with increasing the age of patients. CB in female, married, unemployed/ homemaker, and low economic status caregivers was significantly higher. The level of CB decreased with increasing level of education, the number of caregivers, and declining duration of care.

## Future Direction

It is recommended that future studies investigate the role of supportive and psychosocial interventions on CB and patients' NPS. Also, more extensive studies on patients with mild symptoms in the community are recommended.

## Data Availability Statement

The original contributions presented in the study are included in the article/supplementary material, further inquiries can be directed to the corresponding authors.

## Ethics Statement

This study was approved by Ethical Committee of Guilan University of Medical Sciences. The patients/participants provided their written informed consent to participate in this study.

## Author Contributions

Study design and drafting manuscript: BI, EA, FE-S, and RS. Gathering data: EA. Data analysis: BI and EA. All authors contributed to the article and approved the submitted version.

## Funding

This study was the result of a MD (medical doctor) thesis approved by Guilan University of Medical Sciences.

## Conflict of Interest

The authors declare that the research was conducted in the absence of any commercial or financial relationships that could be construed as a potential conflict of interest.

## Publisher's Note

All claims expressed in this article are solely those of the authors and do not necessarily represent those of their affiliated organizations, or those of the publisher, the editors and the reviewers. Any product that may be evaluated in this article, or claim that may be made by its manufacturer, is not guaranteed or endorsed by the publisher.

## Abbreviations

NPS: Neuropsychiatric symptoms
CB: Caregiver burden
AD: Alzheimer's disease
NPI-Q: Neuropsychiatric Inventory Questionnaire
CBI: Caregiver Burden Inventory
AMB: Aberrant motor behavior
MCI: Mild cognitive impairment
ANOVA: Analysis of variance
AchEs: Acetylcholinesterase inhibitors
SSRIs: Selective serotonin reuptake inhibitors.
